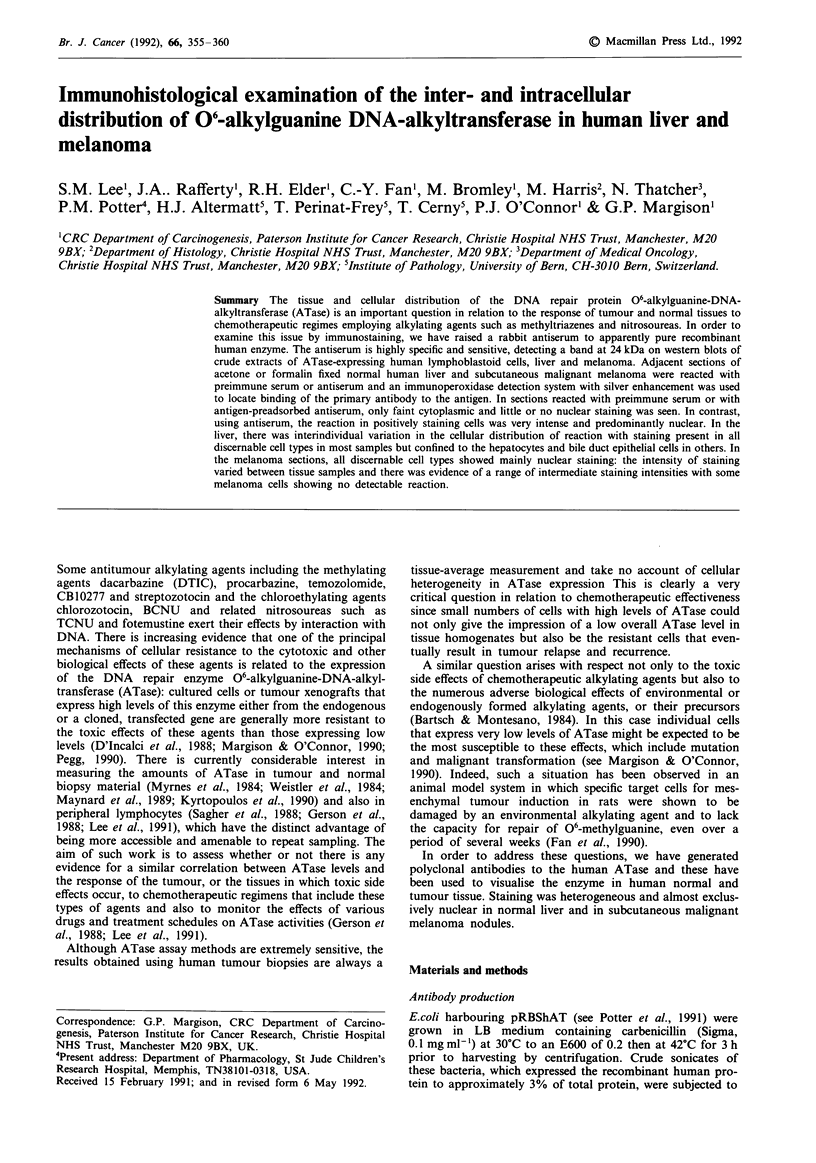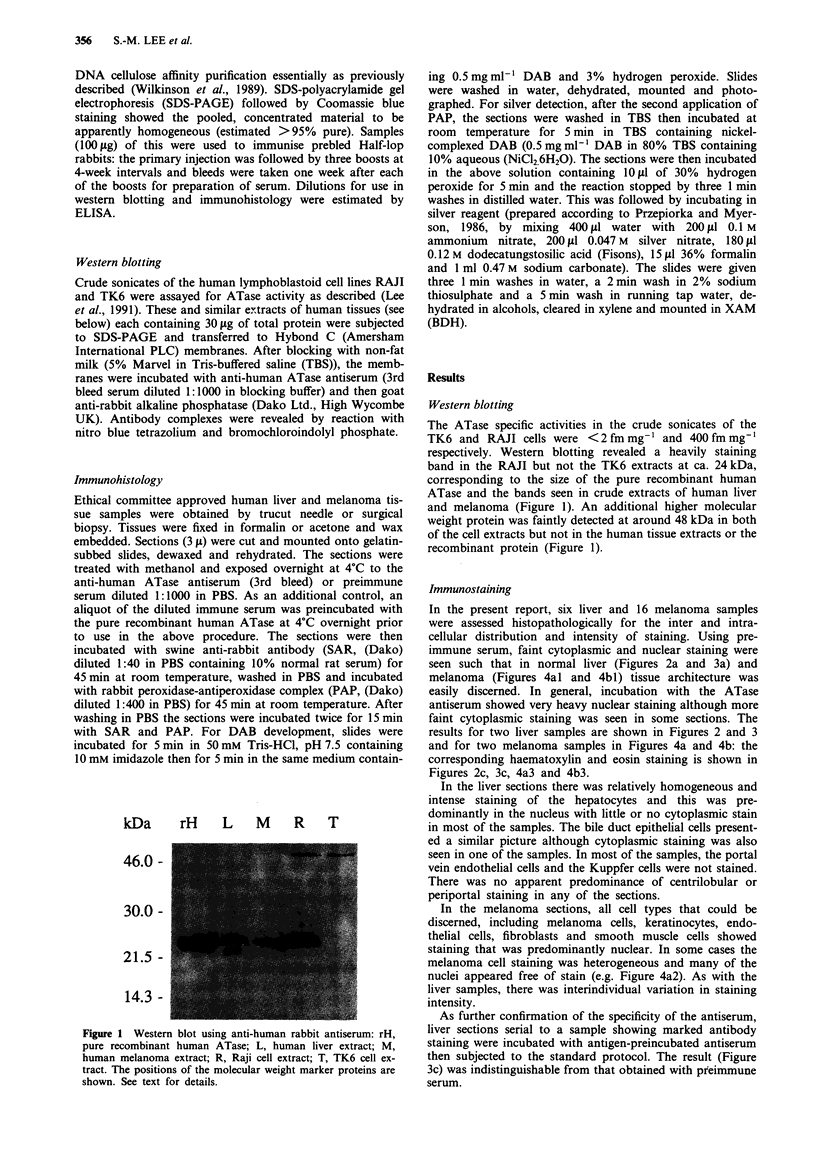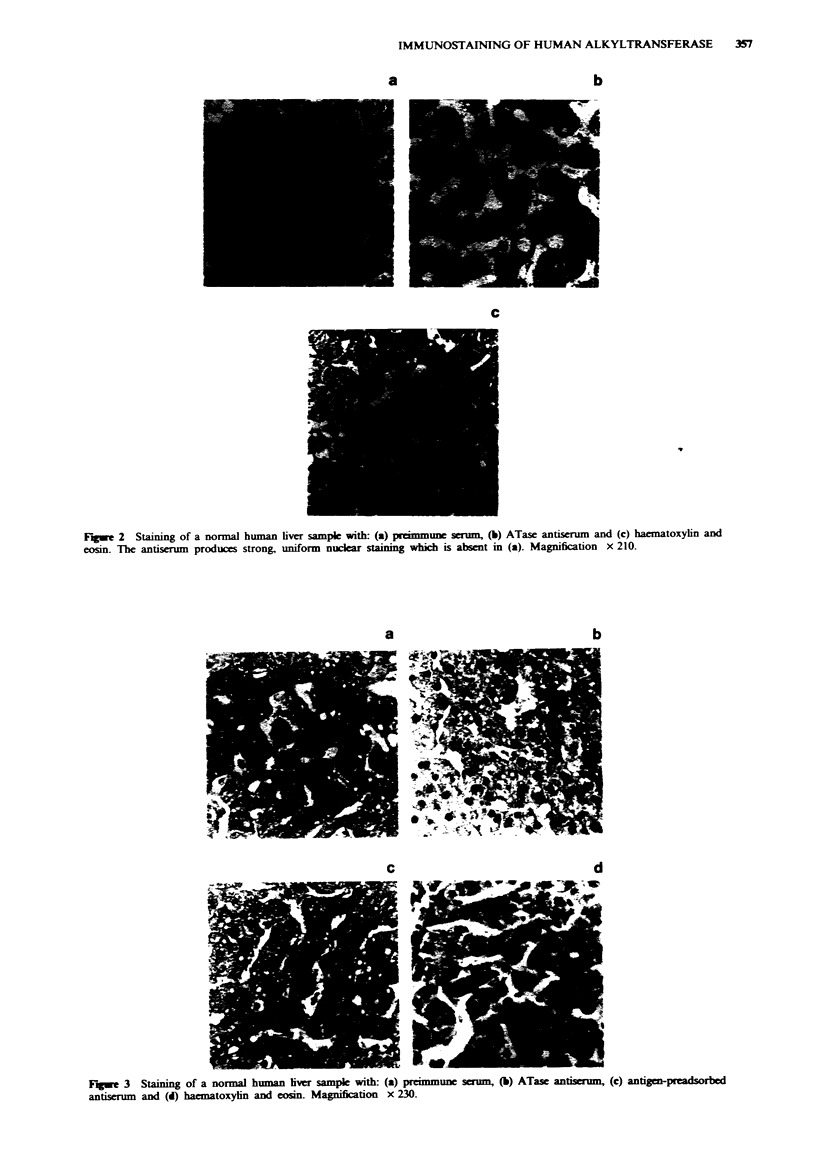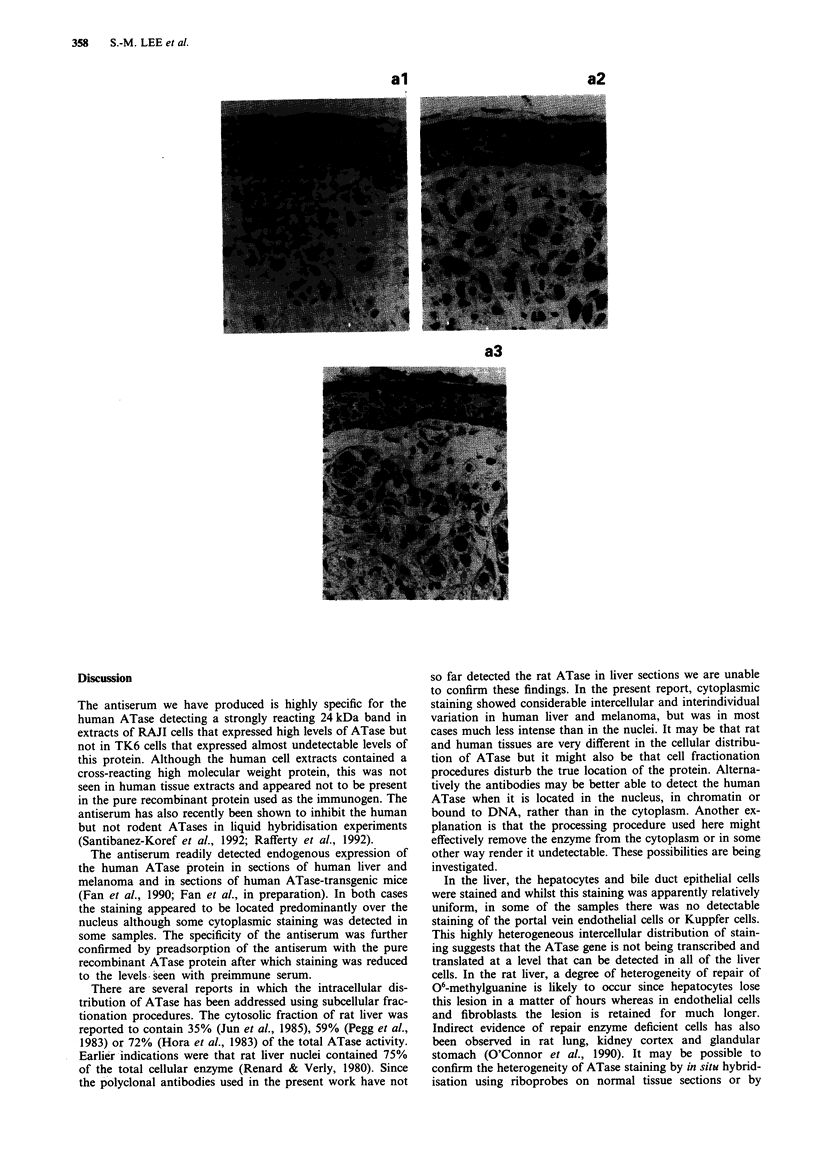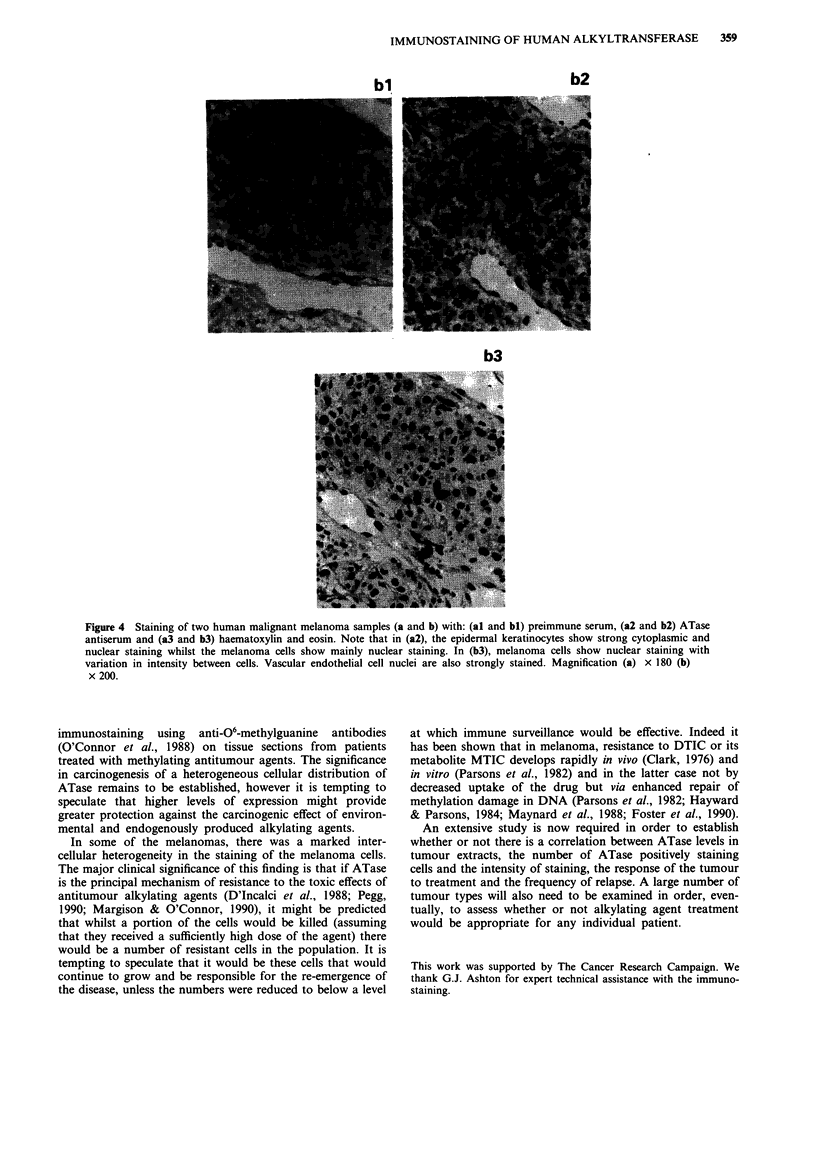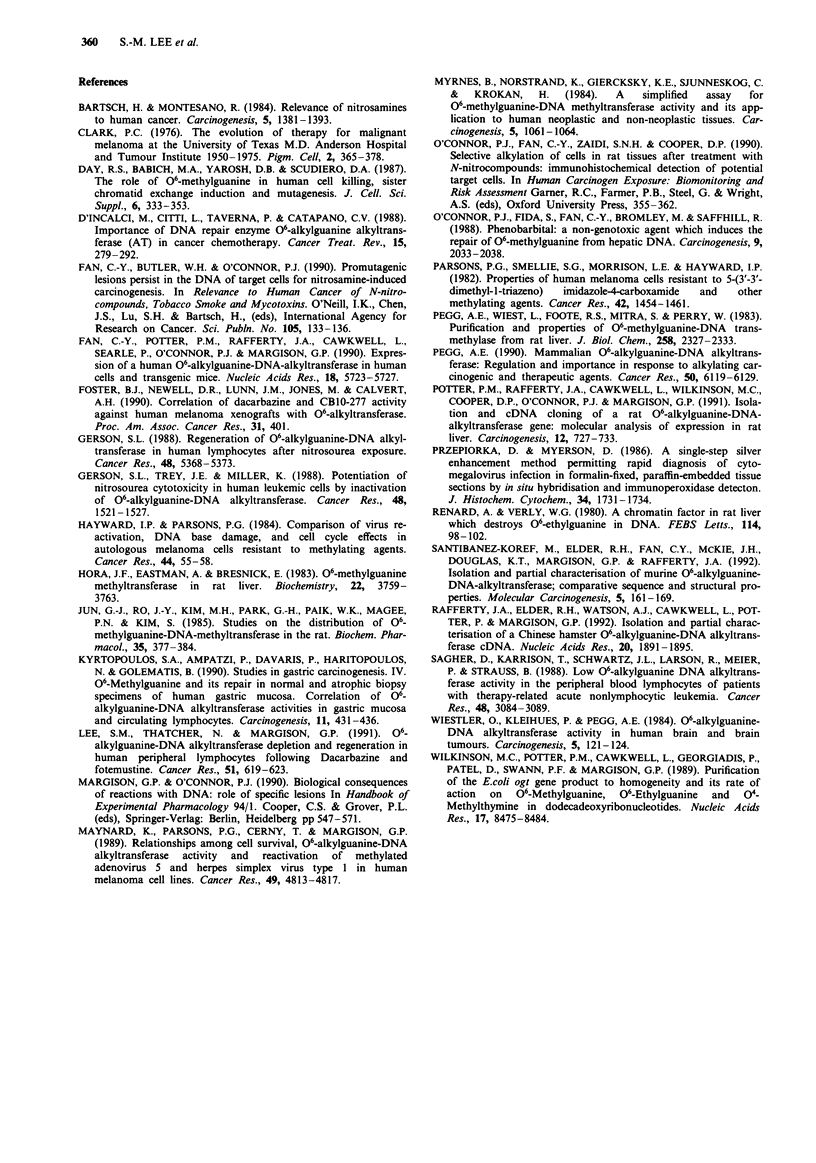# Immunohistological examination of the inter- and intracellular distribution of O6-alkylguanine DNA-alkyltransferase in human liver and melanoma.

**DOI:** 10.1038/bjc.1992.270

**Published:** 1992-08

**Authors:** S. M. Lee, J. A. Rafferty, R. H. Elder, C. Y. Fan, M. Bromley, M. Harris, N. Thatcher, P. M. Potter, H. J. Altermatt, T. Perinat-Frey

**Affiliations:** CRC Department of Carcinogenesis, Paterson Institute for Cancer Research, Christie Hospital NHS Trust, Manchester, UK.

## Abstract

**Images:**


Br. J. Cancer (1992), 66, 355–360 © Macmillan Press Ltd., 1992

### Immunohistological examination of the inter- and intracellular distribution of $O^6$-alkylguanine DNA-alkyltransferase in human liver and melanoma

S.M. Lee[1], J.A.. Rafferty[1], R.H. Elder[1], C.-Y. Fan[1], M. Bromley[1], M. Harris[2], N. Thatcher[3], P.M. Potter[4], H.J. Altermatt[5], T. Perinat-Frey[5], T. Cerny[5], P.J. O'Connor[1] & G.P. Margison[1]

[1]*CRC Department of Carcinogenesis, Paterson Institute for Cancer Research, Christie Hospital NHS Trust, Manchester, M20 9BX;* [2]*Department of Histology, Christie Hospital NHS Trust, Manchester, M20 9BX;* [3]*Department of Medical Oncology, Christie Hospital NHS Trust, Manchester, M20 9BX;* [5]*Institute of Pathology, University of Bern, CH-3010 Bern, Switzerland.*

**Summary** The tissue and cellular distribution of the DNA repair protein $O^6$-alkylguanine-DNA-alkyltransferase (ATase) is an important question in relation to the response of tumour and normal tissues to chemotherapeutic regimes employing alkylating agents such as methyltriazenes and nitrosoureas. In order to examine this issue by immunostaining, we have raised a rabbit antiserum to apparently pure recombinant human enzyme. The antiserum is highly specific and sensitive, detecting a band at 24 kDa on western blots of crude extracts of ATase-expressing human lymphoblastoid cells, liver and melanoma. Adjacent sections of acetone or formalin fixed normal human liver and subcutaneous malignant melanoma were reacted with preimmune serum or antiserum and an immunoperoxidase detection system with silver enhancement was used to locate binding of the primary antibody to the antigen. In sections reacted with preimmune serum or with antigen-preadsorbed antiserum, only faint cytoplasmic and little or no nuclear staining was seen. In contrast, using antiserum, the reaction in positively staining cells was very intense and predominantly nuclear. In the liver, there was interindividual variation in the cellular distribution of reaction with staining present in all discernable cell types in most samples but confined to the hepatocytes and bile duct epithelial cells in others. In the melanoma sections, all discernable cell types showed mainly nuclear staining: the intensity of staining varied between tissue samples and there was evidence of a range of intermediate staining intensities with some melanoma cells showing no detectable reaction.

Some antitumour alkylating agents including the methylating agents dacarbazine (DTIC), procarbazine, temozolomide, CB10277 and streptozotocin and the chloroethylating agents chlorozotocin, BCNU and related nitrosoureas such as TCNU and fotemustine exert their effects by interaction with DNA. There is increasing evidence that one of the principal mechanisms of cellular resistance to the cytotoxic and other biological effects of these agents is related to the expression of the DNA repair enzyme $O^6$-alkylguanine-DNA-alkyltransferase (ATase): cultured cells or tumour xenografts that express high levels of this enzyme either from the endogenous or a cloned, transfected gene are generally more resistant to the toxic effects of these agents than those expressing low levels (D'Incalci *et al.*, 1988; Margison & O'Connor, 1990; Pegg, 1990). There is currently considerable interest in measuring the amounts of ATase in tumour and normal biopsy material (Myrnes *et al.*, 1984; Weistler *et al.*, 1984; Maynard *et al.*, 1989; Kyrtopoulos *et al.*, 1990) and also in peripheral lymphocytes (Sagher *et al.*, 1988; Gerson *et al.*, 1988; Lee *et al.*, 1991), which have the distinct advantage of being more accessible and amenable to repeat sampling. The aim of such work is to assess whether or not there is any evidence for a similar correlation between ATase levels and the response of the tumour, or the tissues in which toxic side effects occur, to chemotherapeutic regimens that include these types of agents and also to monitor the effects of various drugs and treatment schedules on ATase activities (Gerson *et al.*, 1988; Lee *et al.*, 1991).

Although ATase assay methods are extremely sensitive, the results obtained using human tumour biopsies are always a tissue-average measurement and take no account of cellular heterogeneity in ATase expression This is clearly a very critical question in relation to chemotherapeutic effectiveness since small numbers of cells with high levels of ATase could not only give the impression of a low overall ATase level in tissue homogenates but also be the resistant cells that eventually result in tumour relapse and recurrence.

A similar question arises with respect not only to the toxic side effects of chemotherapeutic alkylating agents but also to the numerous adverse biological effects of environmental or endogenously formed alkylating agents, or their precursors (Bartsch & Montesano, 1984). In this case individual cells that express very low levels of ATase might be expected to be the most susceptible to these effects, which include mutation and malignant transformation (see Margison & O'Connor, 1990). Indeed, such a situation has been observed in an animal model system in which specific target cells for mesenchymal tumour induction in rats were shown to be damaged by an environmental alkylating agent and to lack the capacity for repair of $O^6$-methylguanine, even over a period of several weeks (Fan *et al.*, 1990).

In order to address these questions, we have generated polyclonal antibodies to the human ATase and these have been used to visualise the enzyme in human normal and tumour tissue. Staining was heterogeneous and almost exclusively nuclear in normal liver and in subcutaneous malignant melanoma nodules.

### Materials and methods

### *Antibody production*

*E.coli* harbouring pRBShAT (see Potter *et al.*, 1991) were grown in LB medium containing carbenicillin (Sigma, 0.1 mg $ml^{-1}$) at 30°C to an E600 of 0.2 then at 42°C for 3 h prior to harvesting by centrifugation. Crude sonicates of these bacteria, which expressed the recombinant human protein to approximately 3% of total protein, were subjected to

Correspondence: G.P. Margison, CRC Department of Carcinogenesis, Paterson Institute for Cancer Research, Christie Hospital NHS Trust, Manchester M20 9BX, UK.
[4]Present address: Department of Pharmacology, St Jude Children's Research Hospital, Memphis, TN38101-0318, USA.
Received 15 February 1991; and in revised form 6 May 1992.

DNA cellulose affinity purification essentially as previously described (Wilkinson et al., 1989). SDS-polyacrylamide gel electrophoresis (SDS-PAGE) followed by Coomassie blue staining showed the pooled, concentrated material to be apparently homogeneous (estimated >95% pure). Samples (100 µg) of this were used to immunise prebled Half-lop rabbits: the primary injection was followed by three boosts at 4-week intervals and bleeds were taken one week after each of the boosts for preparation of serum. Dilutions for use in western blotting and immunohistology were estimated by ELISA.

### *Western blotting*

Crude sonicates of the human lymphoblastoid cell lines RAJI and TK6 were assayed for ATase activity as described (Lee et al., 1991). These and similar extracts of human tissues (see below) each containing 30 µg of total protein were subjected to SDS-PAGE and transferred to Hybond C (Amersham International PLC) membranes. After blocking with non-fat milk (5% Marvel in Tris-buffered saline (TBS)), the membranes were incubated with anti-human ATase antiserum (3rd bleed serum diluted 1:1000 in blocking buffer) and then goat anti-rabbit alkaline phosphatase (Dako Ltd., High Wycombe UK). Antibody complexes were revealed by reaction with nitro blue tetrazolium and bromochloroindolyl phosphate.

### *Immunohistology*

Ethical committee approved human liver and melanoma tissue samples were obtained by trucut needle or surgical biopsy. Tissues were fixed in formalin or acetone and wax embedded. Sections (3 µ) were cut and mounted onto gelatin-subbed slides, dewaxed and rehydrated. The sections were treated with methanol and exposed overnight at 4°C to the anti-human ATase antiserum (3rd bleed) or preimmune serum diluted 1:1000 in PBS. As an additional control, an aliquot of the diluted immune serum was preincubated with the pure recombinant human ATase at 4°C overnight prior to use in the above procedure. The sections were then incubated with swine anti-rabbit antibody (SAR, (Dako) diluted 1:40 in PBS containing 10% normal rat serum) for 45 min at room temperature, washed in PBS and incubated with rabbit peroxidase-antiperoxidase complex (PAP, (Dako) diluted 1:400 in PBS) for 45 min at room temperature. After washing in PBS the sections were incubated twice for 15 min with SAR and PAP. For DAB development, slides were incubated for 5 min in 50 mM Tris-HCl, pH 7.5 containing 10 mM imidazole then for 5 min in the same medium containing 0.5 mg ml$^{-1}$ DAB and 3% hydrogen peroxide. Slides were washed in water, dehydrated, mounted and photographed. For silver detection, after the second application of PAP, the sections were washed in TBS then incubated at room temperature for 5 min in TBS containing nickel-complexed DAB (0.5 mg ml$^{-1}$ DAB in 80% TBS containing 10% aqueous ($NiCl_2.6H_2O$). The sections were then incubated in the above solution containing 10 µl of 30% hydrogen peroxide for 5 min and the reaction stopped by three 1 min washes in distilled water. This was followed by incubating in silver reagent (prepared according to Przepiorka and Myerson, 1986, by mixing 400 µl water with 200 µl 0.1 M ammonium nitrate, 200 µl 0.047 M silver nitrate, 180 µl 0.12 M dodecatungstosilic acid (Fisons), 15 µl 36% formalin and 1 ml 0.47 M sodium carbonate). The slides were given three 1 min washes in water, a 2 min wash in 2% sodium thiosulphate and a 5 min wash in running tap water, dehydrated in alcohols, cleared in xylene and mounted in XAM (BDH).

**Figure 1** Western blot using anti-human rabbit antiserum: rH, pure recombinant human ATase; L, human liver extract; M, human melanoma extract; R, Raji cell extract; T, TK6 cell extract. The positions of the molecular weight marker proteins are shown. See text for details.

### Results

### *Western blotting*

The ATase specific activities in the crude sonicates of the TK6 and RAJI cells were <2 fm mg$^{-1}$ and 400 fm mg$^{-1}$ respectively. Western blotting revealed a heavily staining band in the RAJI but not the TK6 extracts at ca. 24 kDa, corresponding to the size of the pure recombinant human ATase and the bands seen in crude extracts of human liver and melanoma (Figure 1). An additional higher molecular weight protein was faintly detected at around 48 kDa in both of the cell extracts but not in the human tissue extracts or the recombinant protein (Figure 1).

### *Immunostaining*

In the present report, six liver and 16 melanoma samples were assessed histopathologically for the inter and intracellular distribution and intensity of staining. Using preimmune serum, faint cytoplasmic and nuclear staining were seen such that in normal liver (Figures 2a and 3a) and melanoma (Figures 4a1 and 4b1) tissue architecture was easily discerned. In general, incubation with the ATase antiserum showed very heavy nuclear staining although more faint cytoplasmic staining was seen in some sections. The results for two liver samples are shown in Figures 2 and 3 and for two melanoma samples in Figures 4a and 4b: the corresponding haematoxylin and eosin staining is shown in Figures 2c, 3c, 4a3 and 4b3.

In the liver sections there was relatively homogeneous and intense staining of the hepatocytes and this was predominantly in the nucleus with little or no cytoplasmic stain in most of the samples. The bile duct epithelial cells presented a similar picture although cytoplasmic staining was also seen in one of the samples. In most of the samples, the portal vein endothelial cells and the Kuppfer cells were not stained. There was no apparent predominance of centrilobular or periportal staining in any of the sections.

In the melanoma sections, all cell types that could be discerned, including melanoma cells, keratinocytes, endothelial cells, fibroblasts and smooth muscle cells showed staining that was predominantly nuclear. In some cases the melanoma cell staining was heterogeneous and many of the nuclei appeared free of stain (e.g. Figure 4a2). As with the liver samples, there was interindividual variation in staining intensity.

As further confirmation of the specificity of the antiserum, liver sections serial to a sample showing marked antibody staining were incubated with antigen-preincubated antiserum then subjected to the standard protocol. The result (Figure 3c) was indistinguishable from that obtained with preimmune serum.

**Figure 2** Staining of a normal human liver sample with: (**a**) preimmune serum, (**b**) ATase antiserum and (**c**) haematoxylin and eosin. The antiserum produces strong, uniform nuclear staining which is absent in (**a**). Magnification ×210.

**Figure 3** Staining of a normal human liver sample with: (**a**) preimmune serum, (**b**) ATase antiserum, (**c**) antigen-preadsorbed antiserum and (**d**) haematoxylin and eosin. Magnification ×230.

### Discussion

The antiserum we have produced is highly specific for the human ATase detecting a strongly reacting 24 kDa band in extracts of RAJI cells that expressed high levels of ATase but not in TK6 cells that expressed almost undetectable levels of this protein. Although the human cell extracts contained a cross-reacting high molecular weight protein, this was not seen in human tissue extracts and appeared not to be present in the pure recombinant protein used as the immunogen. The antiserum has also recently been shown to inhibit the human but not rodent ATases in liquid hybridisation experiments (Santibanez-Koref *et al.*, 1992; Rafferty *et al.*, 1992).

The antiserum readily detected endogenous expression of the human ATase protein in sections of human liver and melanoma and in sections of human ATase-transgenic mice (Fan *et al.*, 1990; Fan *et al.*, in preparation). In both cases the staining appeared to be located predominantly over the nucleus although some cytoplasmic staining was detected in some samples. The specificity of the antiserum was further confirmed by preadsorption of the antiserum with the pure recombinant ATase protein after which staining was reduced to the levels seen with preimmune serum.

There are several reports in which the intracellular distribution of ATase has been addressed using subcellular fractionation procedures. The cytosolic fraction of rat liver was reported to contain 35% (Jun *et al.*, 1985), 59% (Pegg *et al.*, 1983) or 72% (Hora *et al.*, 1983) of the total ATase activity. Earlier indications were that rat liver nuclei contained 75% of the total cellular enzyme (Renard & Verly, 1980). Since the polyclonal antibodies used in the present work have not so far detected the rat ATase in liver sections we are unable to confirm these findings. In the present report, cytoplasmic staining showed considerable intercellular and interindividual variation in human liver and melanoma, but was in most cases much less intense than in the nuclei. It may be that rat and human tissues are very different in the cellular distribution of ATase but it might also be that cell fractionation procedures disturb the true location of the protein. Alternatively the antibodies may be better able to detect the human ATase when it is located in the nucleus, in chromatin or bound to DNA, rather than in the cytoplasm. Another explanation is that the processing procedure used here might effectively remove the enzyme from the cytoplasm or in some other way render it undetectable. These possibilities are being investigated.

In the liver, the hepatocytes and bile duct epithelial cells were stained and whilst this staining was apparently relatively uniform, in some of the samples there was no detectable staining of the portal vein endothelial cells or Kuppfer cells. This highly heterogeneous intercellular distribution of staining suggests that the ATase gene is not being transcribed and translated at a level that can be detected in all of the liver cells. In the rat liver, a degree of heterogeneity of repair of $O^6$-methylguanine is likely to occur since hepatocytes lose this lesion in a matter of hours whereas in endothelial cells and fibroblasts the lesion is retained for much longer. Indirect evidence of repair enzyme deficient cells has also been observed in rat lung, kidney cortex and glandular stomach (O'Connor *et al.*, 1990). It may be possible to confirm the heterogeneity of ATase staining by *in situ* hybridisation using riboprobes on normal tissue sections or by

**Figure 4** Staining of two human malignant melanoma samples (**a** and **b**) with: (**a1** and **b1**) preimmune serum, (**a2** and **b2**) ATase antiserum and (**a3** and **b3**) haematoxylin and eosin. Note that in (**a2**), the epidermal keratinocytes show strong cytoplasmic and nuclear staining whilst the melanoma cells show mainly nuclear staining. In (**b3**), melanoma cells show nuclear staining with variation in intensity between cells. Vascular endothelial cell nuclei are also strongly stained. Magnification (**a**) × 180 (**b**) × 200.

immunostaining using anti-$O^6$-methylguanine antibodies (O'Connor *et al.*, 1988) on tissue sections from patients treated with methylating antitumour agents. The significance in carcinogenesis of a heterogeneous cellular distribution of ATase remains to be established, however it is tempting to speculate that higher levels of expression might provide greater protection against the carcinogenic effect of environmental and endogenously produced alkylating agents.

In some of the melanomas, there was a marked intercellular heterogeneity in the staining of the melanoma cells. The major clinical significance of this finding is that if ATase is the principal mechanism of resistance to the toxic effects of antitumour alkylating agents (D'Incalci *et al.*, 1988; Pegg, 1990; Margison & O'Connor, 1990), it might be predicted that whilst a portion of the cells would be killed (assuming that they received a sufficiently high dose of the agent) there would be a number of resistant cells in the population. It is tempting to speculate that it would be these cells that would continue to grow and be responsible for the re-emergence of the disease, unless the numbers were reduced to below a level at which immune surveillance would be effective. Indeed it has been shown that in melanoma, resistance to DTIC or its metabolite MTIC develops rapidly *in vivo* (Clark, 1976) and *in vitro* (Parsons *et al.*, 1982) and in the latter case not by decreased uptake of the drug but *via* enhanced repair of methylation damage in DNA (Parsons *et al.*, 1982; Hayward & Parsons, 1984; Maynard *et al.*, 1988; Foster *et al.*, 1990).

An extensive study is now required in order to establish whether or not there is a correlation between ATase levels in tumour extracts, the number of ATase positively staining cells and the intensity of staining, the response of the tumour to treatment and the frequency of relapse. A large number of tumour types will also need to be examined in order, eventually, to assess whether or not alkylating agent treatment would be appropriate for any individual patient.

This work was supported by The Cancer Research Campaign. We thank G.J. Ashton for expert technical assistance with the immunostaining.

### References

BARTSCH, H. & MONTESANO, R. (1984). Relevance of nitrosamines to human cancer. *Carcinogenesis*, **5**, 1381–1393.

CLARK, P.C. (1976). The evolution of therapy for malignant melanoma at the University of Texas M.D. Anderson Hospital and Tumour Institute 1950–1975. *Pigm. Cell*, **2**, 365–378.

DAY, R.S., BABICH, M.A., YAROSH, D.B. & SCUDIERO, D.A. (1987). The role of $O^6$-methylguanine in human cell killing, sister chromatid exchange induction and mutagenesis. *J. Cell. Sci. Suppl.*, **6**, 333–353.

D'INCALCI, M., CITTI, L., TAVERNA, P. & CATAPANO, C.V. (1988). Importance of DNA repair enzyme $O^6$-alkylguanine alkyltransferase (AT) in cancer chemotherapy. *Cancer Treat. Rev.*, **15**, 279–292.

FAN, C.-Y., BUTLER, W.H. & O'CONNOR, P.J. (1990). Promutagenic lesions persist in the DNA of target cells for nitrosamine-induced carcinogenesis. In *Relevance to Human Cancer of N-nitrocompounds, Tobacco Smoke and Mycotoxins*. O'Neill, I.K., Chen, J.S., Lu, S.H. & Bartsch, H., (eds), International Agency for Research on Cancer. *Sci. Publn. No.* **105**, 133–136.

FAN, C.-Y., POTTER, P.M., RAFFERTY, J.A., CAWKWELL, L., SEARLE, P., O'CONNOR, P.J. & MARGISON, G.P. (1990). Expression of a human $O^6$-alkylguanine-DNA-alkyltransferase in human cells and transgenic mice. *Nucleic Acids Res.*, **18**, 5723–5727.

FOSTER, B.J., NEWELL, D.R., LUNN, J.M., JONES, M. & CALVERT, A.H. (1990). Correlation of dacarbazine and CB10-277 activity against human melanoma xenografts with $O^6$-alkyltransferase. *Proc. Am. Assoc. Cancer Res.*, **31**, 401.

GERSON, S.L. (1988). Regeneration of $O^6$-alkylguanine-DNA alkyltransferase in human lymphocytes after nitrosourea exposure. *Cancer Res.*, **48**, 5368–5373.

GERSON, S.L., TREY, J.E. & MILLER, K. (1988). Potentiation of nitrosourea cytotoxicity in human leukemic cells by inactivation of $O^6$-alkylguanine-DNA alkyltransferase. *Cancer Res.*, **48**, 1521–1527.

HAYWARD, I.P. & PARSONS, P.G. (1984). Comparison of virus reactivation, DNA base damage, and cell cycle effects in autologous melanoma cells resistant to methylating agents. *Cancer Res.*, **44**, 55–58.

HORA, J.F., EASTMAN, A. & BRESNICK, E. (1983). $O^6$-methylguanine methyltransferase in rat liver. *Biochemistry*, **22**, 3759–3763.

JUN, G.-J., RO, J.-Y., KIM, M.H., PARK, G.-H., PAIK, W.K., MAGEE, P.N. & KIM, S. (1985). Studies on the distribution of $O^6$-methylguanine-DNA-methyltransferase in the rat. *Biochem. Pharmacol.*, **35**, 377–384.

KYRTOPOULOS, S.A., AMPATZI, P., DAVARIS, P., HARITOPOULOS, N. & GOLEMATIS, B. (1990). Studies in gastric carcinogenesis. IV. $O^6$-Methylguanine and its repair in normal and atrophic biopsy specimens of human gastric mucosa. Correlation of $O^6$-alkylguanine-DNA alkyltransferase activities in gastric mucosa and circulating lymphocytes. *Carcinogenesis*, **11**, 431–436.

LEE, S.M., THATCHER, N. & MARGISON, G.P. (1991). $O^6$-alkylguanine-DNA alkyltransferase depletion and regeneration in human peripheral lymphocytes following Dacarbazine and fotemustine. *Cancer Res.*, **51**, 619–623.

MARGISON, G.P. & O'CONNOR, P.J. (1990). Biological consequences of reactions with DNA: role of specific lesions In *Handbook of Experimental Pharmacology* 94/1. Cooper, C.S. & Grover, P.L. (eds), Springer-Verlag: Berlin, Heidelberg pp 547–571.

MAYNARD, K., PARSONS, P.G., CERNY, T. & MARGISON, G.P. (1989). Relationships among cell survival, $O^6$-alkylguanine-DNA alkyltransferase activity and reactivation of methylated adenovirus 5 and herpes simplex virus type 1 in human melanoma cell lines. *Cancer Res.*, **49**, 4813–4817.

MYRNES, B., NORSTRAND, K., GIERCKSKY, K.E., SJUNNESKOG, C. & KROKAN, H. (1984). A simplified assay for $O^6$-methylguanine-DNA methyltransferase activity and its application to human neoplastic and non-neoplastic tissues. *Carcinogenesis*, **5**, 1061–1064.

O'CONNOR, P.J., FAN, C.-Y., ZAIDI, S.N.H. & COOPER, D.P. (1990). Selective alkylation of cells in rat tissues after treatment with *N*-nitrocompounds: immunohistochemical detection of potential target cells. In *Human Carcinogen Exposure: Biomonitoring and Risk Assessment* Garner, R.C., Farmer, P.B., Steel, G. & Wright, A.S. (eds), Oxford University Press, 355–362.

O'CONNOR, P.J., FIDA, S., FAN, C.-Y., BROMLEY, M. & SAFFHILL, R. (1988). Phenobarbital: a non-genotoxic agent which induces the repair of $O^6$-methylguanine from hepatic DNA. *Carcinogenesis*, **9**, 2033–2038.

PARSONS, P.G., SMELLIE, S.G., MORRISON, L.E. & HAYWARD, I.P. (1982). Properties of human melanoma cells resistant to 5-(3′-3′-dimethyl-1-triazeno) imidazole-4-carboxamide and other methylating agents. *Cancer Res.*, **42**, 1454–1461.

PEGG, A.E., WIEST, L., FOOTE, R.S., MITRA, S. & PERRY, W. (1983). Purification and properties of $O^6$-methylguanine-DNA transmethylase from rat liver. *J. Biol. Chem.*, **258**, 2327–2333.

PEGG, A.E. (1990). Mammalian $O^6$-alkylguanine-DNA alkyltransferase: Regulation and importance in response to alkylating carcinogenic and therapeutic agents. *Cancer Res.*, **50**, 6119–6129.

POTTER, P.M., RAFFERTY, J.A., CAWKWELL, L., WILKINSON, M.C., COOPER, D.P., O'CONNOR, P.J. & MARGISON, G.P. (1991). Isolation and cDNA cloning of a rat $O^6$-alkylguanine-DNA-alkyltransferase gene: molecular analysis of expression in rat liver. *Carcinogenesis*, **12**, 727–733.

PRZEPIORKA, D. & MYERSON, D. (1986). A single-step silver enhancement method permitting rapid diagnosis of cytomegalovirus infection in formalin-fixed, paraffin-embedded tissue sections by *in situ* hybridisation and immunoperoxidase detecton. *J. Histochem. Cytochem.*, **34**, 1731–1734.

RENARD, A. & VERLY, W.G. (1980). A chromatin factor in rat liver which destroys $O^6$-ethylguanine in DNA. *FEBS Letts.*, **114**, 98–102.

SANTIBANEZ-KOREF, M., ELDER, R.H., FAN, C.Y., MCKIE, J.H., DOUGLAS, K.T., MARGISON, G.P. & RAFFERTY, J.A. (1992). Isolation and partial characterisation of murine $O^6$-alkylguanine-DNA-alkyltransferase; comparative sequence and structural properties. *Molecular Carcinogenesis*, **5**, 161–169.

RAFFERTY, J.A., ELDER, R.H., WATSON, A.J., CAWKWELL, L., POTTER, P. & MARGISON, G.P. (1992). Isolation and partial characterisation of a Chinese hamster $O^6$-alkylguanine-DNA alkyltransferase cDNA. *Nucleic Acids Res.*, **20**, 1891–1895.

SAGHER, D., KARRISON, T., SCHWARTZ, J.L., LARSON, R., MEIER, P. & STRAUSS, B. (1988). Low $O^6$-alkylguanine DNA alkyltransferase activity in the peripheral blood lymphocytes of patients with therapy-related acute nonlymphocytic leukemia. *Cancer Res.*, **48**, 3084–3089.

WIESTLER, O., KLEIHUES, P. & PEGG, A.E. (1984). $O^6$-alkylguanine-DNA alkyltransferase activity in human brain and brain tumours. *Carcinogenesis*, **5**, 121–124.

WILKINSON, M.C., POTTER, P.M., CAWKWELL, L., GEORGIADIS, P., PATEL, D., SWANN, P.F. & MARGISON, G.P. (1989). Purification of the *E.coli ogt* gene product to homogeneity and its rate of action on $O^6$-Methylguanine, $O^6$-Ethylguanine and $O^4$-Methylthymine in dodecadeoxyribonucleotides. *Nucleic Acids Res.*, **17**, 8475–8484.